# A Snapshot of Clinical Psychology Trainees’ Exposure to Parenting Approaches and Attitudes Toward Child Rights

**DOI:** 10.1177/13591045261466511

**Published:** 2026-07-02

**Authors:** Elisa Romano, Sarah Zak

**Affiliations:** 1 School of Psychology, 6363 University of Ottawa, Ottawa, ON, Canada

**Keywords:** clinical psychology, parenting, parent behaviour management, positive parenting, child rights, coursework, supervision

## Abstract

**Purpose:**

Clinical psychology graduate trainees may pursue training in child, youth, and family services, which often encompasses the delivery of parenting interventions. Two broad and fundamentally different parenting approaches are Parent Behaviour Management and Positive Parenting. We examined the types of parenting approaches to which child/family clinical psychology trainees were exposed, along with their attitudes toward child rights and punitive parenting.

**Major Findings:**

We collected data for 73 Canadian doctoral students. For the subset of 54 child/family trainees, about one-third were not exposed to any parenting programs in clinical supervision or coursework. The remaining students learned about programs that fell within a Parent Behaviour Management approach only or within both Parent Behaviour Management and Positive Parenting. The majority (90.4%) of the larger sample were not familiar with the UN Convention on the Rights of the Child, although their child rights attitudes were highly favourable and associated with lower punitive parenting approval.

**Conclusions:**

These preliminary findings suggest that clinical psychology trainees are learning about the two broad parenting approaches. However, they do not have much training in children’s rights, which is concerning given the associations with punitive parenting attitudes and the importance of centering children’s perspectives in clinical work with parents.

## Plain Language Summary

Why was the study done? Clinical psychologists specializing in children and families often work with parents having difficulties parenting their child. There are two broad and different parenting approaches. One is called parent behaviour management, and it focuses more attention on changing children’s behaviours. The other is called positive parenting, and it focuses more attention on parents’ behaviours and how they can improve the relationship with their child. We were interested in understanding which approach graduate students were learning. We also wanted to know whether students were learning about children’s rights.

What did the researchers do? We contacted Canadian universities and asked them to send information about the study to graduate students in clinical psychology. Those who agreed to participate completed a questionnaire online that asked about different things like their training experiences and children’s rights.

What did the researchers find? We received information from 73 students, with 54 of them saying that they were learning to do clinical work with children and families. For these 54, about 1 in 3 had not learned about any parenting approaches. For the other students, some said that they only learned about parent behaviour management, and the others said that they learned about both approaches. For all 73 students, 9 in 10 said that they had never heard about the United Nations Convention on the Rights of the Child, but they agreed that children have rights. The more students agreed that children have rights, the more they agreed that children should not be punished by parents.

What do the findings mean? Some students in clinical psychology have not learned about how to work with parents, but it could be that they did not yet have a chance. The other students learned about both types of parenting approaches, although more just learned about parent behaviour management. Almost all students had not learned about children’s rights.

In Canada, data from 2023 indicate that there were 21,337 registered clinical psychologists across the ten provinces and three territories (Canadian Institute for Health Information, 2025). There is no publicly available information on these psychologists’ scope of practice, but a significant portion engages in psychological services with children, youth, and families. Since approximately 1 in 5 children and youth experience mental health challenges, such as anxiety, depression, behavioural difficulties, and attention-deficit/hyperactivity disorder (Bor et al., 2014; Leeb et al., 2024), the psychological services offered can be broad in nature. In some instances, services may include not only the young person but also parents through evidence-based, parent-focused interventions (e.g., Baan et al., 2025). Such interventions address concerns around the child-parent relationship and parenting approaches that be impacting the well-being of the child or youth.

## Parenting Approaches

One common approach to challenging child-parent situations prioritizes changing children’s behaviours through learning principles aimed at either increasing parent-desired behaviours or decreasing undesired behaviours through a system of rewards and non-physical punishments. Common interventions include Parent-Child Interaction Therapy (Querido et al., 2002), Triple P (Sanders et al., 2014), Incredible Years (Webster-Stratton, 2007), 1-2-3 Magic (Phelan, 2010), and the MATCH (Modular Approach to Therapy for Children with Anxiety, Depression, Trauma or Conduct Problems) Protocol (Chorpita & Weisz, 2009). Practices within a *Parent Behaviour Management* approach aimed at decreasing undesired behaviours might include taking away objects (e.g., toys, phones) or activities (e.g., playing a videogame, going to the playground) that are meaningful for a child, planned ignoring of a child, or administration of a time-out. The goal of punishment is to make a child’s behaviour less likely to occur by parents either introducing a consequence, such as a time-out or removing a toy/activity, or not providing the child with something, such as attention (Holden et al., 2017).

Time-out from positive reinforcement is a punishment where “a child’s access to rewards, usually parental attention, is temporarily removed contingent upon a problem behavior and reinstated following a specified period of nonproblem behavior” (Dadds & Tully, 2019, p. 795). Time-out is one of various parenting practices included in recommendations by the Canadian Paediatric Society (Williams et al., 2019). Dadds and Tully (2019) note that time-out can be conceptualized and effectively implemented not only within learning theory but also within theories of attachment, self-regulation, and family systems. Other scholars (Durrant, 2020; Durrant et al., 2020; Holden et al., 2022) would argue against this statement. Holden et al. (2022) stated that a time-out fails “to provide a forum for communication. Importantly, by distancing the child from the parent, it does not promote warmth or dyadic connections” (p. 245).

A second approach to addressing challenging parenting situations can be labelled *Positive Parenting*, where practices prioritize the child-parent relationship and, specifically, communication, mentorship, and collaborative conflict resolution (Durrant, 2016; Holden et al., 2022; Seay et al., 2014). Within this approach, for example, a parent would remain physically close to their child following a challenging situation (e.g., time-in) to help with regulation, identify underlying needs, and engage in teaching and problem solving (Holden et al., 2022). The Canadian Paediatric Association (Williams et al., 2019) also encourages time-ins as one of various parenting practices and highlights the importance of prioritizing the child-parent relationship, understanding what might be going on for a child in difficult moments, helping a child manage their feelings, and guiding a child in problem solving. It is stated that, with time-in, “the caregiver invites the child to sit and talk about their feelings and behaviour in an age-appropriate way. Time-in emphasizes connection and comfort. Preferential use of time-ins and other positive parenting strategies should not necessarily preclude selective, targeted use of time-outs for specific misbehaviours in children over 3 years old” (Williams et al., 2019, p. 345). Examples of positive parenting interventions include the Whole-Brain Child (Siegel & Bryson, 2012), Emotion Coaching (Ramsden & Hubbard, 2002), Positive Discipline in Everyday Parenting (Durrant, 2016), Collaborative and Proactive Problem Solving (Booker et al., 2016; Greene, 2011), Circle of Security (Hoffman et al., 2017), and Tuning in to Kids (Havighurst et al., 2013). These interventions do not include time-out and would constitute what Holden et al. (2017) have labelled to be “strong” forms of positive parenting. This is in contrast to “lite” approaches that remain based on behaviour management principles to shape children’s behaviour through the use, for example, of time-outs as a non-physical punishment (e.g., Triple P, Incredible Years; Holden et al., 2017).

## Punishment and Children’s Rights

The Parent Behaviour Management and Positive Parenting approaches were developed from two fundamentally different starting points, with the former focusing on a child’s behaviour and the latter focusing on a parent’s behaviour. One commonality is the rejection of child physical punishment, which is likely based on the solid single-study and meta-analytic research base attesting to physical punishment’s detrimental short- and long-term impacts on children’s development and well-being (e.g., Afifi et al., 2017; Gershoff, 2002; Gershoff & Grogan-Kaylor, 2016; Ward et al., 2023). There are programs developed within the Positive Parenting approach, such as Positive Discipline in Everyday Parenting (Durrant, 2016), that explicitly mention physical punishment’s violation of children’s rights to protection from all forms of “physical or mental violence” and reference the United Nations Convention on the Rights of the Child (United Nations General Assembly, 2006).

One can argue that upholding children’s rights to protection from all forms of harm and to participation in all matters that affect them needs to be at the core of all parenting work in which child and family psychologists engage and, as such, needs to be included in the graduate training of clinical psychology students. This is important because there is research, albeit limited, showing that child rights and child punishment attitudes are related. Gallitto et al. (2021) examined the association between knowledge and attitudes toward children’s rights and attitudes toward child spanking (as the most common form of physical punishment) among 329 Canadian undergraduate psychology students. Results showed that more favourable attitudes toward children’s rights predicted less favourable attitudes toward spanking. Knowledge of children’s rights served as a moderator in that greater knowledge strengthened the impact of child rights attitudes on spanking attitudes.

## Training Experiences and Needs

Although we did not locate any similar past research, several studies have more broadly assessed the learning experiences of students pursuing specialized training in child and adolescent clinical psychology. In Pidano et al.’s (2010) survey of training directors from 29 doctoral North American programs that offered child and adolescent training, there were questions about 17 key training domains (including parent and family interventions). For parent interventions, 38% of programs required exposure (i.e., introduction to the domain via a seminar or observational learning), 28% required experience (i.e., practice), and 14% required expertise (i.e., course work and extensive practice to attain competence). For family interventions, 45% of programs required student exposure, 28% required experience, and 3% required expertise. For parent and family interventions, there was more frequent mention of Parent Behaviour Management training, Multisystemic Therapy, and Parent-Child Interaction Therapy, the latter of which is “commonly used with younger children with behavioral problems and involves components both to modify and build parent-child relationships and to reduce noncompliant and maladaptive behaviors” (p. 125).

In a later study, Pidano and Whitcomb (2012) conducted a similar study from the perspective of 227 U.S. graduate students pursuing child and adolescent clinical training. The most endorsed theoretical orientation was cognitive-behavioural (47.5%), followed by integrative approaches (41.63%), behavioural (5.43%), family systems (2.26%), interpersonal (0.9%), psychodynamic/object relations (0.45%), and humanistic-existential (0.45%). For parent interventions, 6.5% of students reported having achieved exposure, 42% achieved experience, and 47.5% achieved expertise. For family interventions, 23.6% reported having achieved exposure, 47.2% achieved experience, and 21.6% achieved expertise. As interventions, students mentioned Parent Behaviour Management training, family therapy, and attachment-based therapy, although there were no data on the exact interventions and strategies being used.

Finally, a recent study by Stough et al. (2024) examined data from training directors of 100 primarily U.S.-based doctoral, internship, or postdoctoral programs that offered training in clinical child and adolescent psychology. The training experiences offered by doctoral programs in terms of coursework on interventions for this population ranged from 50–100%, depending on the opportunity level (e.g., exposure vs. emphasis). There was no detailed information on course types or interventions available to students. For training gaps, 43.6% of doctoral training directors indicated that students could be better prepared for working with children, parents, and families, and 46.2% reported better preparation around child development.

## Study Objectives

Parenting interventions often are an important component of the work undertaken by clinical psychologists who specialize with children and families. There are two fundamentally different approaches that have different treatment targets, with Parent Behaviour Management focusing more on children’s behaviours and Positive Parenting placing greater emphasis on the behaviours of parents and the child-parent relationship (Holden et al., 2017). The attention to children’s rights may figure more or less prominently within these two parenting approaches.

Our first study objective was to describe the parenting approaches and programs about which clinical psychology trainees in Canada learn. Specifically, we aimed to describe recommended parenting practices and goals within the Parent Behaviour Management and Positive Parenting approaches. As part of their graduate studies, clinical psychology students pursuing child, youth, and family training would likely learn about different parenting approaches that presumably would then inform their parenting work during training and after the completion of their graduate program. In fact, clinical psychology doctoral programs must meet certain training standards to be accredited by the Canadian Psychology Association, including functional competencies in assessment and intervention (https://cpa.ca/accreditation/resources/).

The second objective was to examine trainees’ child rights knowledge and attitudes as we believe a child rights perspective is important to parenting work that centers children’s developmental needs and their perspectives on clinical interventions in which they are involved. We also examined any links between knowledge/attitudes toward children’s rights and attitudes toward punitive parenting practices. In line with a previous Canadian study with undergraduate students (Gallitto et al., 2021), we hypothesized that more favourable attitudes toward children’s rights would be associated with less approval of child punishment. Study findings, while preliminary, were expected to provide valuable information on the types of parenting approaches that future clinical psychologists might bring to their work with families, which undoubtedly impacts the well-being of children and youth.

## Methods

### Study Design

A cross-sectional design was used in which trainees completed standardized measures at a single time point. Trainees also provided responses to three hypothetical and challenging parenting situations; however, this qualitative information was not analyzed in the current study because of space limitations and will be considered in a future study. The current study had a large descriptive component, and there also was an examination of the way in which child rights knowledge and attitudes (i.e., predictor variables) might impact attitudes toward child physical and non-physical punishment (i.e., outcome variables).

### Participants and Procedures

Following approval from our university’s office of research ethics and integrity (File # H-04-22-7987), we posted study information on social media and contacted the program directors of accredited Canadian clinical psychology programs to distribute a study email to enrolled graduate students (after obtaining ethics approval from each university). The recruitment period was from Spring 2022 – Spring 2023. The recruitment information specified that the study was exploring the parenting approaches to which students were exposed as part of their training and their views of various parenting practices and children’s rights. Inclusion criteria were enrollment in an accredited clinical psychology program in Canada and the ability to understand English as all study materials were in this language. Students could click on a link to the online consent form and questionnaire, which was about 20 minutes long. At the end of the study, students could open another electronic window in the Qualtrics platform to provide an email address for a gift card draw. The sample initially consisted of 100 students across 16 universities. However, 27 were removed because of a large amount of missing data (>50%), leaving a final sample of 73 who participated in this cross-sectional online survey design (see Figure 1).

**Figure 1.**
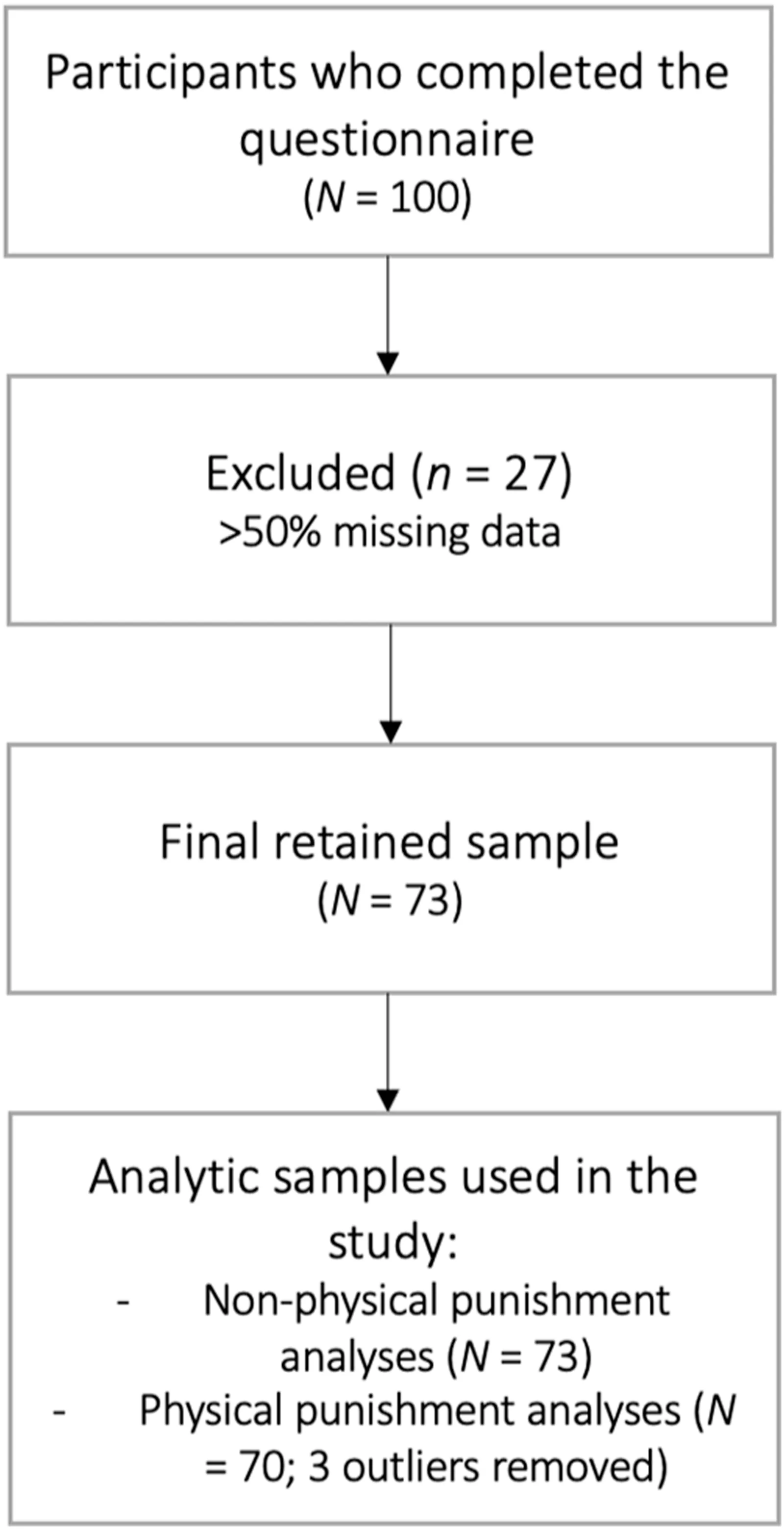
Participant flow from initial sample to analytic study samples

### Measures

The questionnaire had various sections:

#### Socio-Demographics and Training Information

Data were collected on trainees’ socio-demographics and on their training, such as the type of graduate program in which they were enrolled and their year in the program. For trainees who indicated working (or planning to work) clinically with children and families, there was an additional set of questions about completed assessment and intervention hours, parenting programs presented in coursework and clinical supervision, parenting practices they might recommend (e.g., time-out, emotion coaching), and treatment goals they would prioritize (e.g., improve children’s behaviour, improve parental well-being).

#### Attitudes Toward Child Punishment

The Positive Discipline in Everyday Parenting Questionnaire (Gallitto & Romano, 2024) was used to assess attitudes toward physical and non-physical punishment. There are 9 items for physical punishment, such as *It’s OK to spank a 5-year old’s bottom if they do something dangerous* and *Spanking or slapping is fine as long as the parent is not angry.* Each item is rated on a 6-point scale from 1 (strongly disagree) to 6 (strongly agree). Scores could range from 9-54, with higher scores indicating greater approval. The internal consistency for the whole sample was α = .87. For non-physical punishment, there are 7 items, such as *If a 3-year old won’t share their toys, they should be made to sit on a chair for 3 minutes* and *If two teenage siblings won’t stop fighting, their parent should threaten to take something away, such as their phones*. Each item is rated on a 6-point scale from 1 (strongly disagree) to 6 (strongly agree). Scores could range from 7-42, with higher scores indicating greater approval. The internal consistency for the whole sample was α = .76.

#### Child Rights Knowledge

The What I Know About the United Nations Convention on the Rights of the Child scale (Campbell & Covell, 2001) was used to assess knowledge about children’s rights. There are 16 statements answered with either *true, false, or I don’t know*. Examples include *According to the CRC [Convention on the Rights of the Child], parents have to allow time for children to engage in play and leisure* and *According to the CRC, parents are allowed to spank their children if their religion supports this practice.* A correct response receives 2 points, an incorrect response receives 0 points, and 1 point is given to a response of I don’t know. Scores could range from 0-32, with higher scores indicating greater knowledge.

#### Child Rights Attitudes

The How I Feel About Rights for Others measure (Covell & Howe, 2001), was used and specifically, 13 of the 30 items that focused on children’s rights. Examples include *right of children never to be spanked by adults, regardless of what they do or say* and *right of children to have a say in all decisions that affect them, even if adults think they are too inexperienced.* Each item is rated on a 7-point scale from 1 (not at all important) to 7 (extremely important). Scores could range from 13-91, with higher scores indicating more favourable attitudes. There was good internal consistency for the whole sample (α = .80).

#### Hypothetical Challenging Parenting Situations

Child and family trainees were presented with three hypothetical and typical challenging parenting situations and asked to provide ideas on how they might approach this situation clinically with a parent. The situations were developed by a Canadian developmental-clinical psychologist with extensive child development and positive parenting expertise, in consultation with the current study’s second author and with several colleagues. The hypothetical parenting situations were created as part of data collection for another project in which we evaluated a positive parenting program, using parents’ responses to the three hypothetical situations. For the current study, the same situations were used but the respondents were trainees, instead of parents. These data were not analyzed for the current study because of space limitations; however, they will be considered in a separate study focused on this qualitative information.

### Data Analyses

For the first objective, we focused on students pursuing child and family clinical work (*n* = 54, 74.0%). Descriptive data were generated to assess their exposure to and perspectives on various parenting approaches, programs, practices, and goals. For the second objective, two separate linear regression analyses were conducted with the whole sample (*N* = 73). Child rights attitudes was entered as the independent variable, and attitudes toward physical and non-physical punishment was examined as separate dependent variables. We could not include child rights knowledge as an independent variable because only seven (9.6%) students indicated having read the United Nations Convention on the Rights of the Child (CRC), although a greater number (*n* = 31, 42.5%) reported having heard of the CRC. Using G*Power, a sensitivity power analysis indicated that the study was adequately powered (90%) to detect medium-sized effects (*f* ^*2*^= 0.15) with a probability level of .05 and one predictor. Variables were screened for multivariate normality, including skew and kurtosis, with cut-off scores set at <+/- 2 (George & Mallery, 2009). The physical punishment attitudes variable had negative skewness and three outliers. The outliers were removed, resulting in a reduced sample size (*n* = 70) for analyses involving this variable. A log transformation was performed to improve the distribution and meet assumptions. Analyses involving the other variables were conducted using the full sample. Descriptive statistics, including frequencies, percentages, means, and standard deviations, were calculated for study variables and sample characteristics. Inferential statistics were also reported using correlations, effect sizes, and associated statistics, where relevant. Analyses were conducted in SPSS 30. The data and analytic material (i.e., data coding and statistics) can be made available upon request. Note that only the quantitative measures were analyzed in the current study. The qualitative data from trainees’ responses to the hypothetical parenting situations were not included and will form the basis for a future study.

## Results

### Sample Description

Trainees represented 16 universities, with most coming from the province of Ontario (57.5%), followed by Manitoba (13.7%), Alberta (6.8%), Saskatchewan (6.8%), New Brunswick (5.5%), British Columbia (4.1%), Nova Scotia (2.8%), and prefer not to respond (2.8%). The majority identified as female (90%), followed by male (10%), and the average age was 27.5 years (*SD* = 3.5, range = 23-44). Most trainees identified as White (75.3%), followed by Middle Eastern (5.5%), Indigenous (4.1%), East Asian (4.1%), Black (2.7%), South and Southeast Asian (1.4%), Hispanic (1.4%), and prefer to self-describe (5.5%; Indo-Caribbean, Jewish, >1 background). The majority were enrolled in a doctoral program (80.8%), followed by a master’s program (19.2%). Trainees were spread across different years: Year 1 (21.1%); Year 2 (26.8%); Year 3 (16.9%); Year 4 (18.3%); and Year 5+ (16.9%). The majority were in pre-internship (84.9%), followed by internship year (9.6%) and post-internship year (5.5%).

### Student Training Experiences (Objective 1)

#### Approaches and Programs

The sub-sample (*n* = 54) pursuing child and family training reported an average of 123.6 supervised assessment hours (*SD* = 136.9, range = 0-500) and 223.4 supervised intervention hours (*SD* = 435.4, range = 0-2,500). Training hours were not significantly associated with physical or non-physical punishment attitudes (*p* > .05). Presented with a list, trainees indicated that they worked primarily within a cognitive-behavioural orientation (70.4%), followed by integrative (64.8%), humanistic-existential (25.9%), and systemic (22.2%). Students could also specify other theoretical orientations, and the following were listed: emotion-focused (3.8%); acceptance and mindfulness-based (1.9%); and dialectical-behaviour (1.9%). Percentages exceed 100 since students could select more than one orientation.

Figure 2 shows that, for *coursework*, 38.9% reported no training exposure to any of the listed parenting programs. For remaining students, the greatest exposure was to the MATCH Protocol and Incredible Years program (each at 29.6%), followed by Parent-Child Interaction Therapy (24.1%), Circle of Security (18.5%), Emotion Coaching (18.5%), and Triple P (14.8%). There was an option to specify other programs, and trainees listed Emotion-Focused Family Therapy (3.8%), Barkley Model of ADHD (1.9%), and Acceptance and Commitment Therapy (1.9%).

**Figure 2.**
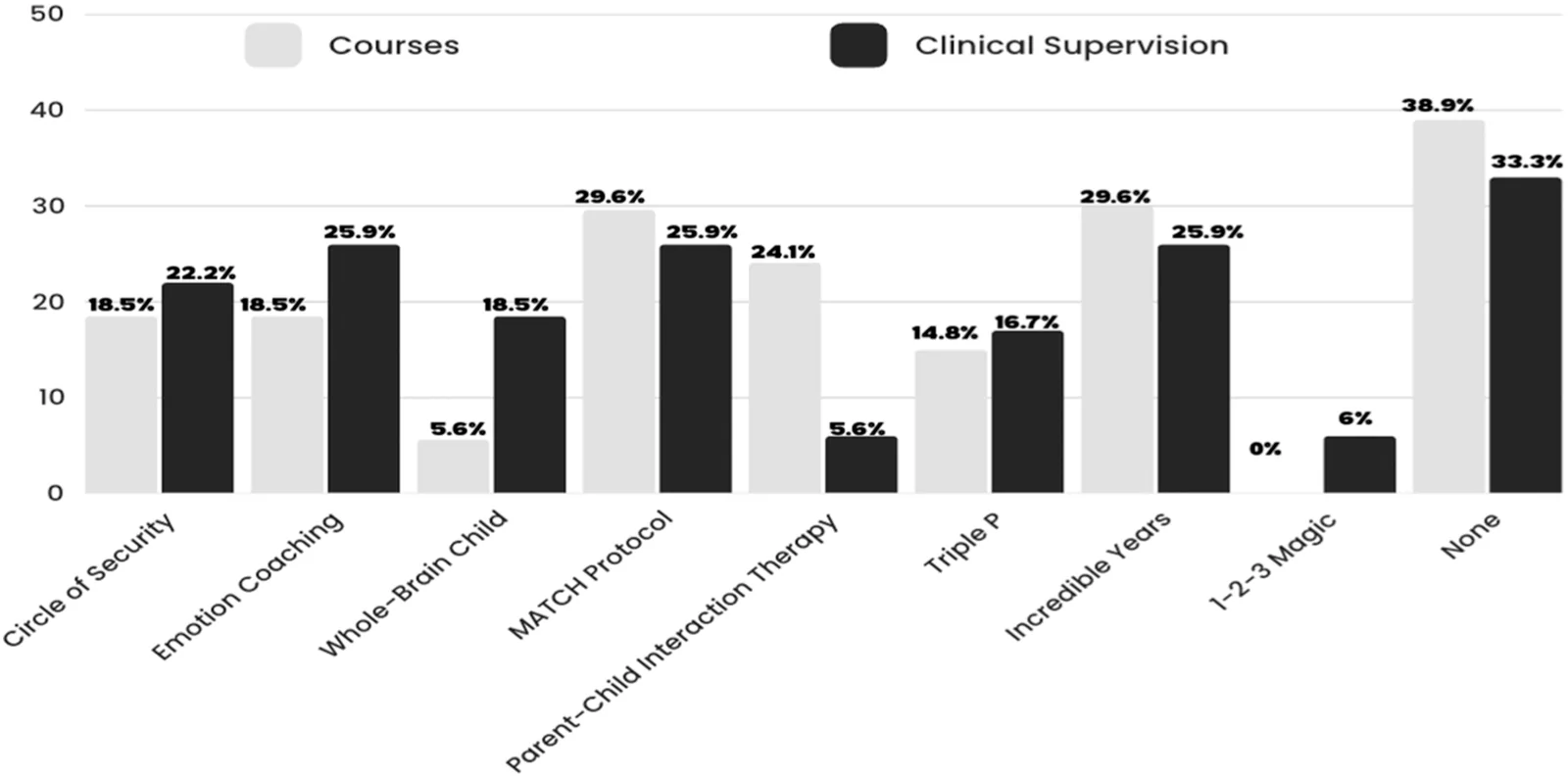
Parenting programs included in coursework and clinical supervision (*N* = 54)

Collapsing the listed programs into the two broad parenting approaches, Table 1 indicates that most students, in their coursework, were either exposed only to Parent Behaviour Management approaches (31%) or to both Parent Behaviour Management and Positive Parenting approaches (28%). For *clinical supervision*, 33.3% indicated no training exposure to any of the listed programs. For remaining students, there was equal exposure to the MATCH Protocol, Incredible Years, and Emotion Coaching (each at 25.9%), followed by Circle of Security (22.2%), Whole-Brain Child (18.5%), and Triple P (16.7%). For the option to specify other programs, trainees listed Emotion-Focused Family Therapy (5.7%), Connect Trauma-Informed and Attachment-Based Parenting Program (3.8%), Watch, Wait, and Wonder (1.9%), Parent Management Training (5.7%), Neurosequential Model of Therapeutics (1.9%), Building Better Behaviours (1.9%), Positive Behaviour Support (1.9%), Building Regulation in Dual Generations (1.9%), and Family Check-Up (1.9%). Table 1 indicates that, during clinical supervision, most students learned about both Parent Behaviour Management and Positive Parenting approaches (28%), followed by only Parent Behaviour Management approaches (22%).

**Table 1.** Parenting Approaches Included in Coursework and Clinical Supervision (*N* = 54)

​	Coursework	Clinical Supervision
Only parent behaviour management	31%	22%
Only positive parenting	2%	17%
Both approaches	28%	28%
No approaches	39%	33%

#### Practices and Goals

Trainees were also presented with a list of eight parenting practices and asked which ones they might recommend when working with parenting challenges. We grouped the practices into the parenting approach with which they were most aligned. Table 2 indicates that the greatest number of responses (80) were for Positive Parenting practices (i.e., time-in followed by emotion coaching), relative to a Parent Behaviour Management approach where there were 60 responses primarily around rewards or “privileges”, followed by consequences and then time-out. Talking and distracting/redirecting can be found in both parenting approaches. Unsurprisingly, many students endorsed the importance of talking with a child, while a smaller number indicated distraction/redirection, and no student would recommend spanking. There was an option to indicate other parenting practices that trainees might recommend, with the responses as follows: validation; praise; involvement of child in developing their reward plan; and parent modelling of emotion regulation for the child.

**Table 2.** Recommended Parenting Practices (*N* = 54)

​	Number of responses	%
Positive parenting approach	80	​
Time-in or similar (e.g., connecting, comforting, hugging)	42	52.5
Emotion coaching	38	47.5
Parent behaviour management approach	60	​
Consequences (e.g., taking away objects or activities)	15	25.0
Time-out	10	16.7
Rewards or “privileges”	35	58.3
Other listed practices	68	​
Talking (e.g., explaining, discussing, problem solving)	45	66.2
Distracting/redirecting	23	33.8
Spanking	0	0

*Note.* Trainees could check as many parenting practices that would apply.

Trainees were presented with a list of seven treatment goals and were asked how important each one would be when working with parents. Table 3 shows that students prioritized all the goals, with means ranging from 5.39 to 6.72. The highest scores went equally to improving parents’ understanding of their child’s needs and improving children’s well-being (*M* = 6.72). The least important goal was around improving the child’s behaviour (*M* = 5.39).

**Table 3.** Importance of Parenting Treatment Goals (*N* = 54)

Treatment goals	Mean	Standard deviation
To improve children’s well-being	6.72	.49
To improve parents’ understanding of their children’s needs	6.72	.49
To improve the parent-child relationship	6.57	.72
To improve children’s self-regulation skills	6.27	.87
To improve parents’ self-regulation skills	5.85	1.37
To improve parents’ well-being	5.52	.93
To improve children’s behaviour	5.39	1.19

*Note.* Possible range from 1-7, with higher scores indicating greater perceived importance.

#### Link Between Child Rights Attitudes and Punitive Parenting Attitudes (Objective 2)

Returning to the full sample of 73 participants, the mean score on attitudes toward children’s rights (*M* = 81.92; *SD* = 6.40) indicated highly favourable attitudes. The mean score for physical punishment (*M* = 10.43; *SD* = 2.14) and non-physical punishment attitudes (*M* = 13.48; *SD* = 4.34) indicated low approval of both punitive parenting practices. The regression analysis for physical punishment (*n* = 70) was significant (*F*(1, 68) = 14.33, *p* < .001) and showed that more favourable attitudes toward children’s rights were associated with lower approval of child physical punishment (*B* = −.15, *SE* = .04, 95% CI [-.23, −.07]). The *R*^*2*^ indicated that 17% of the variance in physical punishment scores could be explained by the child rights attitude variable. The analysis for non-physical punishment was significant (*F*(1, 71) = 9.40, *p* = .003) and showed that more favourable child rights attitudes were associated with lower approval of child non-physical punishment (*B* = −.23, *SE* = .07, 95% CI [-.37, −.08]). The *R*^*2*^ indicated that 12% of the variance in non-physical punishment scores could be explained by the child rights attitude variable.

To examine these relationships in greater detail for the 54 students pursuing child and family training, we ran a correlational analysis with child rights attitudes, physical and non-physical punishment attitudes, and the various parenting practices that students might recommend. Table 4 reveals that more favourable attitudes toward children’s rights were significantly associated with lower approval of child physical (*r* = −.45) and non-physical (*r* = −.28) punishment, mirroring the regression results. There was a positive correlation between non-physical punishment and physical punishment attitudes (*r* = .37). Child rights attitudes, as well as physical punishment attitudes, were not significantly correlated with any of the parenting practices. For non-physical punishment, more favourable attitudes were significantly associated with greater recommended use of consequences (*r* = .25). Among the parenting practices, time-in was not significantly correlated with time-out, but all the other practices were significantly associated with one another. Several of these associations would be expected (e.g., greater recommendation for consequences was positively associated with greater recommendation for rewards/“privileges” and for time-outs; greater recommendation for emotion coaching was positively associated with greater recommended use of time-ins). However, other associations would not be expected, such as greater recommendations for emotion coaching being positively correlated with greater recommended use of time-outs.

**Table 4.** Correlations among child rights attitudes, physical/non-physical punishment attitudes, and parenting practices (N = 54)

​	1	2	3	4	5	6	7	8	9	10
1. Child rights attitudes	-	​	​	​	​	​	​	​	​	​
2. Physical punishment attitudes	−.45**	-	​	​	​	​	​	​	​	​
3. Non-physical punishment attitudes	−.28*	.37**	-	​	​	​	​	​	​	​
4. Time-in	−.04	−.01	.08	-	​	​	​	​	​	​
5. Emotion coaching	−.05	−.07	.07	.54**	-	​	​	​	​	​
6. Consequences	−.06	.17	.25*	.39**	.52*	-	​	​	​	​
7. Time-out	−.03	.12	.13	.23	.27*	.28*	-	​	​	​
8. Rewards or “privileges”	−.22	.05	.16	.62**	.43**	.45**	.40**	-	​	​
9. Talking	−.05	.06	.05	.61**	.63**	.38**	.30*	.55**	-	​
10. Distracting or redirecting	−.06	−.03	.03	.47**	.42**	.57**	.44**	.44**	.50**	-

*p < .05, **p < .01, ***p < .001.

## Discussion

In this study, we were interested in clinical psychology trainees’ exposure to different parenting approaches, as well as their knowledge and attitudes toward children’s rights. To our knowledge, such a study has not been conducted within a Canadian context or elsewhere. These preliminary findings offered valuable information on the types of parenting approaches that future clinical psychologists in Canada might bring to their work with families, which undoubtedly impacts the well-being of children and youth. Moreover, findings on the link between child rights attitudes and child physical/non-physical punishment attitudes offered insights around the importance of child rights training within graduate clinical psychology programs.

For study objectives, we *first* described the training exposure of 54 graduate students seeking child and family clinical training to two parenting approaches, namely Parent Behaviour Management and Positive Parenting, as well as accompanying programs and practices. The majority identified with a cognitive-behavioural (7 in 10) or integrative (6.5 in 10) orientation, which is consistent with past U.S. research using a larger student sample (Pidano & Whitcomb, 2012). About 3-4 in 10 trainees had not been exposed to any parenting programs during coursework or clinical supervision, which perhaps was a function of not yet having had any opportunities because of their more junior program status. However, we were unable to test this hypothesis.

The remaining students had training experience with a range of programs that fit either within a Parent Behaviour Management approach only or within both approaches. For parenting practices that trainees would use clinically, there were a greater number of recommendations aligned with Positive Parenting (i.e., time-in, emotion coaching) than with Parent Behaviour Management (i.e., rewards/privileges, consequences, time-out). Students prioritized a range of parenting treatment goals that focused not only on improving the well-being and self-regulation of children and parents alike but also on improving the child-parent relationship and helping parents better understand their child’s needs. There is no past similar research, but U.S. trainees in Pidano and Whitcomb’s (2012) study also reported experiences with Parent Behaviour Management training, family therapy, and attachment-based therapy, although there were no data on the percentage exposed to these various approaches or to the more specific programs and practices that students were implementing. Nonetheless, our findings show similarities in that students in the current study also endorsed training exposure to both Parent Behaviour Management and Positive Parenting (attachment-based) approaches. Findings from primarily U.S. training directors (Pidano et al., 2010; Stough et al., 2024) show that most programs require some level of exposure to parent and family intervention through coursework or clinical practice. Our findings also indicated that most trainees (60-70%) acquired some training experience with parenting interventions.

Turning to specific parenting programs, past research shows that both students (Pidano & Whitcomb, 2012) and training directors (Pidano et al., 2010) made common reference to Multisystemic Therapy and Parent-Child Interaction Therapy. The current study findings were dissimilar in this respect. There was no mention of Multisystemic Therapy, and exposure to Parent-Child Interaction Therapy was only endorsed by 24.2% of trainees in terms of coursework and 5.6% of trainees during clinical supervision. Differences may be a function of sample features (Canadian versus U.S. sample), training models, study methodologies, and research publication year. Specifically, the Pidano studies are 13-15 years old, and there have been changes to parenting work since then, especially regarding greater exposure to positive parenting approaches. Altogether, the findings from the current study suggest that child and family clinical trainees in Canada learn about the two broad parenting approaches (i.e., Parent Behaviour Management, Positive Parenting), as well as programs and practices that fall within these approaches.

As a *second* objective, we used the full sample of 73 clinical psychology trainees to examine child rights attitudes and links with punitive parenting attitudes. The majority (90.4%) had not heard of or read the United Nations Convention on the Rights of the Child. Nonetheless, they held highly favourable attitudes toward children’s rights, as well as highly unfavourable attitudes toward child physical and non-physical punishment. These findings are encouraging because we believe that knowledge about the rights of children to protection from all forms of harm and to participation in matters that affect them (e.g., their relationship with parents, clinical services) needs to be incorporated in clinical training, both in coursework and clinical supervision. In this way, students can advocate for children’s needs and rights in their clinical work with this population.

Learning about the rights of children also is important because of its link with punitive parenting attitudes. As hypothesized and in line with past research (Gallitto et al., 2021), trainees who held more favourable attitudes toward children’s rights also reported lower approval of child physical and non-physical punishment. The finding for physical punishment is not surprising as there is solid research on its negative child impacts (e.g., Gershoff, 2002; Gershoff & Grogan-Kaylor, 2016), and it is not included in the parenting approaches to which trainees are exposed. For non-physical punishment, the association is important because the use of such practices as time-out is not included in “strong” positive parenting interventions, despite its inclusion in behaviourally based “lite” positive parenting interventions (Holden et al., 2017). Altogether, the findings suggest that one way to move clinical psychology trainees away from favourable punitive parenting views is to teach them about children’s rights; this applies to favourable views about both physical (e.g., spanking) and non-physical (e.g., time-out) punishment. It would have been interesting to examine these associations as a function of students’ training background (e.g., theoretical orientation, program year, number of completed training hours), but the small sample size did not permit such analyses.

Interestingly, findings from the current study did not show any significant correlations between child rights attitudes and various parenting practices. It is possible that these analyses were too detailed in their focus on specific practices; however, we cannot offer any definitive explanation given the small sample size and the lack of past research. The correlational findings also indicated that practices more closely aligned with Positive Parenting were associated with Parent Behaviour Management practices in expected ways (i.e., negatively associated with one another) and unexpected ways (i.e., positively associated with one another). It is possible that trainees took the approach of endorsing many of the practices, because they did not yet have much clinical experience (i.e., 3-4 in 10 had not learned about any parenting programs) and/or because these practices made intuitive sense. It is also possible that, without knowing the specific challenging parenting situation, all trainees opted to select as many practices as possible that could be helpful for children and parents. In this study, trainees who reported having learned about parenting tended to report exposure to practices falling within both Positive Parenting and Parent Behaviour Management approaches; this broad experience might have led to the endorsement of a wide range of parenting practices. In addition, some of the unexpected findings (e.g., emotion coaching recommendations were positively linked with time-out recommendations) might be a function of the fact that “lite” positive parenting programs combine practices aligned with “strong” positive parenting, such as emotion coaching and time-ins, with those from behaviour management approaches that include non-physical punishment, such as consequences and time-outs. These “lite” positive parenting programs, including Incredible Years, Triple P, Parent-Child Interaction Therapy, and MATCH Protocol, were among those to which trainees in the current study were exposed through coursework and clinical supervision.

### Limitations and Future Research Directions

Despite our broad recruitment efforts, the student response to our cross-sectional study was modest. As such, it would be best to consider our findings exploratory in nature and not generalizable to clinical psychology trainees in Canada. In addition, the sample consisted of students who identified primarily as female and White. A larger and more representative sample would be important to make firmer conclusions about trainees’ exposure to different parenting approaches, programs, practices, and goals across Canadian clinical psychology graduate programs. We would also be able to gather a more fulsome understanding of the extent to which information on children’s rights is presented in graduate training as well as the way such knowledge and attitudes can influence perspectives on the use of punitive parenting practices. Although our study was limited to the Canadian context, the topic has international relevance in that child and family clinical psychologists (and mental health practitioners more generally) worldwide may offer parenting services and use programs that fall within the two broad parenting approaches, namely Positive Parenting and Parent Behaviour Management. As such, examining the training that clinical psychology students receive in other countries around parenting approaches and practices may be important.

We gathered important quantitative information from the student survey, and it would be beneficial to explore the findings further by way of a qualitative component (e.g., focus groups, interviews). Questions to explore could include the following: How are students exposed to various parenting programs during coursework and clinical supervision (i.e., what does the training look like)? How do students reconcile their endorsement of a range of parenting practices and goals that fall into parenting approaches that are fundamentally quite different? How do students view time-outs? and What are students’ perceptions around the utility of child rights knowledge in their clinical work? Of course, there would still be the potential limitation associated with convenience sampling in terms of participant self-selection into the study. In other words, it may be that clinical psychology trainees who participate have more favourable attitudes toward children’s rights and/or stronger views about certain parenting approaches or practices.

Finally, we intend to analyze the data collected from trainees’ responses to three hypothetical and typical challenging parenting situations in another study. This qualitative information will help us understand the way trainees interpret the scenarios and suggest various parenting practices within the two broad approaches of Parent Behaviour Management and Positive Parenting. We would also be able to explore the way trainees’ responses are associated with graduate training background and experiences.

### Implications

While our findings are preliminary in nature, they indicate that most Canadian graduate students pursuing child and family clinical training have exposure to the two broad parenting approaches, specifically Parent Behaviour Management and Positive Parenting, along with related programs and practices. However, there is relatively greater exposure to Parent Behaviour Management, either by itself or in combination with Positive Parenting. It appears that trainees would use a range of parenting practices across the two broad approaches, with the more common ones being rewards or “privileges”, time-in, and emotion coaching. Students also seem to prioritize a variety of goals when working with parents experiencing challenging child-parent situations, suggesting that they adopt a comprehensive approach that considers both child and parent well-being along with the attachment relationship. Finally, most trainees were not aware of the United Nations Convention on the Rights of the Child, although attitudes toward children’s rights were highly favourable and were associated with lower approval of physical and non-physical forms of child punishment. Bridging our findings together, from a program training perspective, it seems important to teach graduate students to work clinically with families using parenting approaches, programs, and practices that are aligned with a child rights framework and that center the needs, rights, and viewpoints of children (Romano, 2020).

## Data Availability

The data and analytic material (data coding and statistics) can be made available upon request for verification purposes.*
